# Supplementary material for: Chromothripsis during telomere crisis is independent of NHEJ, and consistent with a replicative origin
Source: Genome Res. 2019 May;29(5):737–49. doi: 10.1101/gr.240705.118 (PMC6499312; doi:10.1101/gr.240705.118)
Supplement: Supplemental Material [file supp_gr.240705.118_Supplemental_file_1.zip › contigs/annotated_contigs/DB51/contig.2.DB51_length_645_mean_cov_15.1782945736.docx]

**DB51_length_645_mean_cov_15.1782945736**

```
AATATATGTGCAATATATATCTGCAAGATGTATTTGCCTACTCTTACTGAGCTTTCTCAATTGAGTACAAGTTTCAGTGCATTTAGTTC
 >chr12:13203847-13204160 + E=1e-176
ATAAATTGTGGTTTGGGAATGTGGCTGTTTCCTATTTTGTGTTCTATTTTTATTTTTCTGTATTTGTTTTCCTGGTTTTAGGGAAGACA

GTGTGACAGAAATGCCCACTTTTGCCATCATTAACTAGAAGCATGTAATCTTGCTAAACAGACCAATCTGACAGCAGCTTTTTTCTCAA

AGAATGCTTCAAATTGTTTTTATAAATTTCTTACAGATTTTATA|AA|AGTACTATTTTGAATTAAGTTTCTAATAATTGATTTATTTT
                                             >chr12:13206000-13206334 + E=3e-171
CTTGTAATTTGTGCCCTTAAAAATCAAGTCTAATATTTATTGGGCAAAATGTATAGTTTTGAAAATAAAAATAAATAGAAATAATTTCT

AGTTATTAGGATGACAGATTATTCAGTATTTTCTTTGGATTTTTTTTGAAAGTACTTAATGTTATAATTCTGTGGAATGTTCATTTTAA

AAAATCTAAAATTTATTGATTTTTTTAATGTACCAAATACTAACTTTAAACATCAAAGGTTTTACTTTCATCATCTGGTATTGGGGGAA

TTAGCCTCCTATATTTCAACGTAG
```
